# Dataset on electrochemical stability and activity of Au-decorated Pt surface for oxygen reduction reaction in acidic media

**DOI:** 10.1016/j.dib.2019.104897

**Published:** 2019-11-27

**Authors:** Young Min Park, Hyun-Jong Kim

**Affiliations:** Surface Technology Group, Korea Institute of Industrial Technology (KITECH), Incheon, 21999, Republic of Korea

**Keywords:** Proton exchange membrane fuel cell, Au–Pt/C, Durability, Electrochemical surface area, Accelerated degradation test

## Abstract

Hydrogen-air proton exchange membrane fuel cells (PEMFC) have been drawn considerable attention as one of clean energy sources for transportation applications. To achieve the long lifetime of PEMFC for the transportation application, it is required to reduce the loss of electrochemical surface area which is known to result from dissolution of Pt nanoparticles and the size change of nanoparticle. Herein, we decorated Au on commercial Pt/C catalyst with various ratio of Au: Pt in a range of 2 to 0.33: 1 using a chemical reduction method with trisodium citrate. X-ray Diffraction (XRD) result clearly shows that the Au are well deposited on the surface of Pt/C catalysts. The electrochemical surface areas of catalyst are assessed as a function of Au concentration potential cycling in accelerated degradation tests. Furthermore, the oxygen reduction reaction (ORR) activity of Au–Pt/C is also estimated in comparison with that of commercial Pt/C using a single cell operation. X-ray photoelectron spectroscopy analysis shows that Au incorporation on Pt/C changes electron density of Pt surface and, consequently more reductive because of difference in work function between Au and Pt. Finally, we provide a series of dataset on the effect of Au on the surface of Pt/C catalyst to stabilize the electrochemical surface area.

**Table:** Specifications Table

Subject area	Energy
More specific subject area	Proton exchange membrane fuel cells
Type of data	Table and figures
How data was acquired	Cyclic voltammetry (IviumStat, Ivium Technologies) X-ray photoelectron spectroscopy (K-Alpha spectrometer, Thermo Fisher Scientific) X-ray diffraction (D8 Discover X-ray diffractometer, Bruker) PEMFC single cell operation (PEMFC station, CNL Energy)
Description of data collection	The crystal sizes of Pt and Au were calculated from the XRD peak of (200) plane using the Scherrer equation. The binding energy of XPS spectrum was corrected by setting the C1s aliphatic signal at 284.8 eV. Electrochemical surface area of catalyst was calculated from cyclic voltammogram. Cyclic voltammogram was obtained between −0.2 or −0.15 and 0.8 V (vs. Ag/AgCl) at a scanning rate of 5 mV s^−1^ in a typical three-electrode cell with oxygen free 0.5 M H_2_SO_4_ aqueous solution. Before the measurement, the potential of the working electrode was cycled for 5 sweeps at 100 mV s^−1^ to electrochemically clean the catalyst surface. The PEMFC performance was measured in a small-scale laboratory cell with an external electrode (2 × 2 cm^2^) by feeding hydrogen and air at 70 °C under 100% relative humidity.
Data format	Raw and analyzed
Data source location	Korea Institute of Industrial Technology (KITECH), Incheon, 21999, Republic of Korea
Data accessibility	The data are with this article.

**Table:** 

**Value of the Data** • These data provide information of the effect of Au on the electrochemical stability of Pt/C catalyst for PEMFC when it is incorporated on the surface of Pt/C catalyst with a various molar ratio of Au to Pt from 0.33:1 to 2:1. • The data can be useful for the PEMFC engineer who should improve the lifetime of electrode for better performance as providing the effect of Au in Pt/C catalyst on the loss of electrochemical surface area. • Au incorporated on the surface of Pt/C catalyst increase the electron density of Pt surface, thus inhibit the dissolution of Pt ion during potential cycling.

## 1 Data

**Fig. 1:**
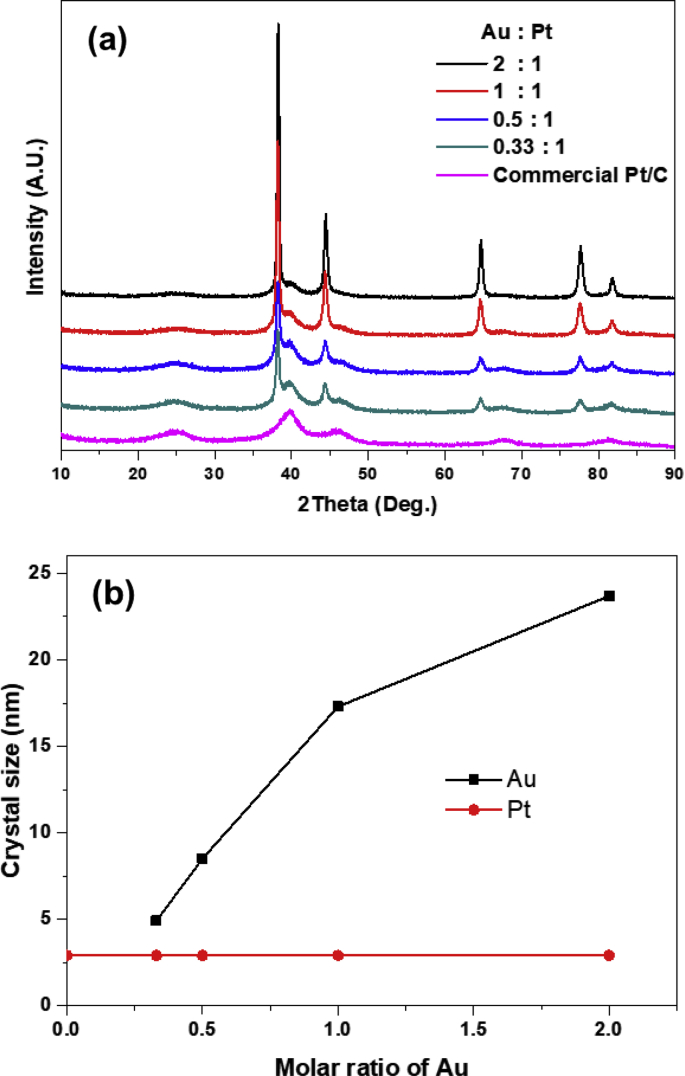
(a) X-ray Diffraction patterns of Au-incorporated Pt/C catalysts with molar ratio of Au to Pt, 2:1, 1:1, 0.5:1, 0.33:1 and commercial Pt/C, respectively and (b) crystal size of Au and Pt with molar ratio of Au in Au–Pt/C.

The loss of electrochemical surface area (ESA) in Pt/C electrode under electrochemical environment have seriously hindered PEMFC from having a long life to be commercialized as an alternative energy sources for transportation [1,2]. To address this problem, many researches have been attempted such as nanoscale coating on Pt surface, development of Pt alloying, and introduction of other materials to prevent dissolution and coalescence of Pt particles [[3], [4], [5]]. Among them, the deposition of Au atom on Pt is reported to have a stabilizing effect on an underlying Pt metal surface under highly oxidizing conditions and suppress Pt dissolution during potential cycling, without decreasing the oxygen reduction reaction (ORR) kinetics [6]. In this work, we compared the stabilizing effect of Au on Pt/C catalyst as a function of Au concentration, thus provide the dataset on the behavior of ESA loss with Au concentration using accelerating degradation test. X-ray diffraction clearly shows that Au are well deposited on Pt/C catalyst via the chemical reduction of chloroauric acid (HAuCl_4_) with sodium citrate at 80 °C (Fig. 1(a)). X-ray diffraction patterns of Au and Pt was indexed into a fcc-type cubic lattice occurring bulk gold and platinum. Depending on the concentration of Pt/C, the molar ratio of Au to Pt is controlled to the range of 2 to 0.33:1. The crystal size of Au based on (220) plane, calculated by Scherrer equation, increases with the concentration of Au up to 23.7nm at the ratio of Au to Pt, 2:1 indicating that the crystal growth of Au occur during reduction of Au ion on the surface of Pt/C catalyst (Fig. 1(b)). The crystal size of Pt calculated with (220) plane, on the other hands, is maintained during deposition of Au, which means the Au incorporation during chemical reduction do not significantly affect on the original microstructure of Pt/C electrode.

**Fig. 2:**
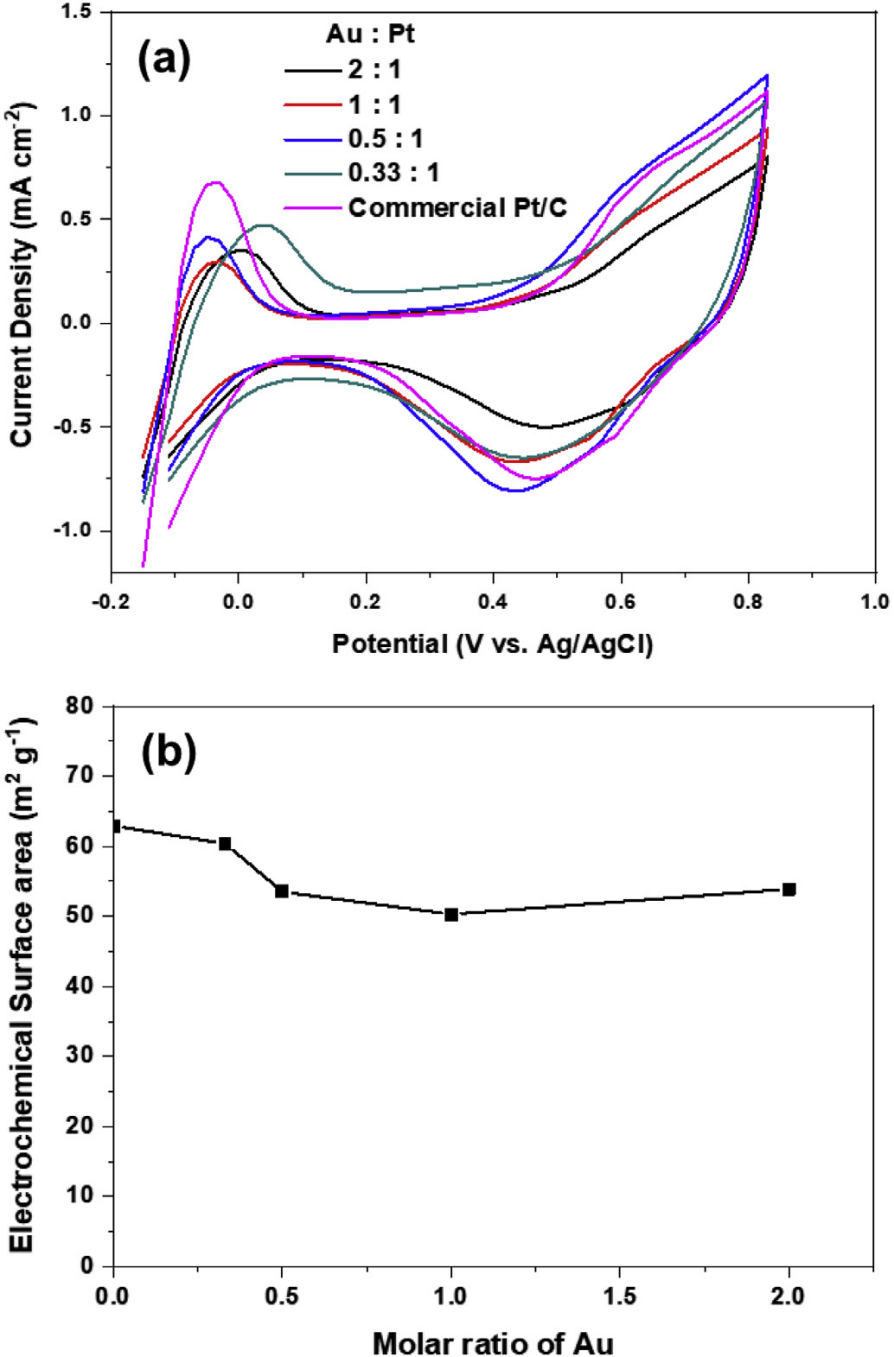
(a) CV curves of Au–Pt/C electrode with a voltage range of −0.2 V–0.8 V and (b) calculated electrochemical surface area with molar ratio of Au in AuPt/C.

**Fig. 3:**
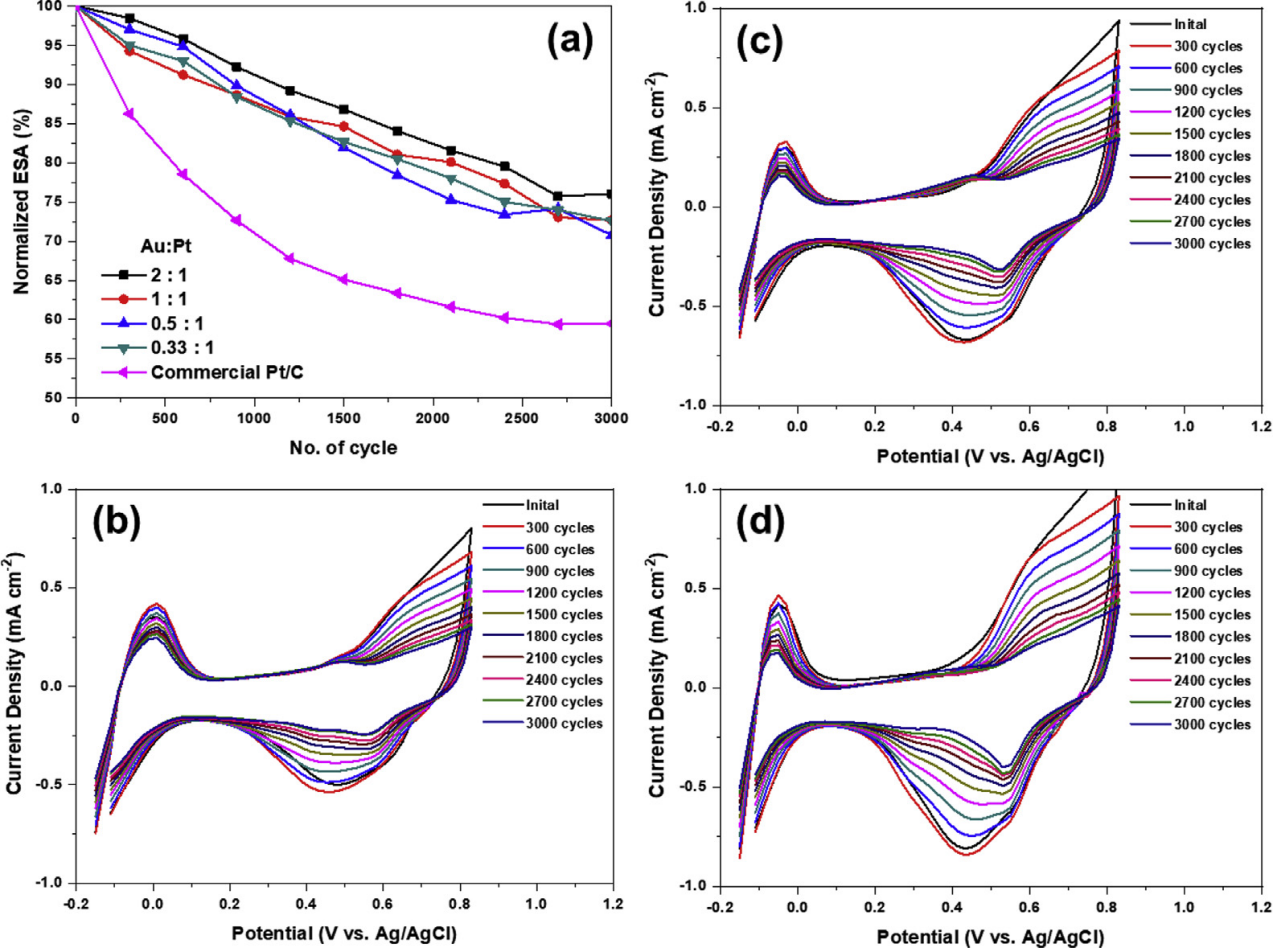

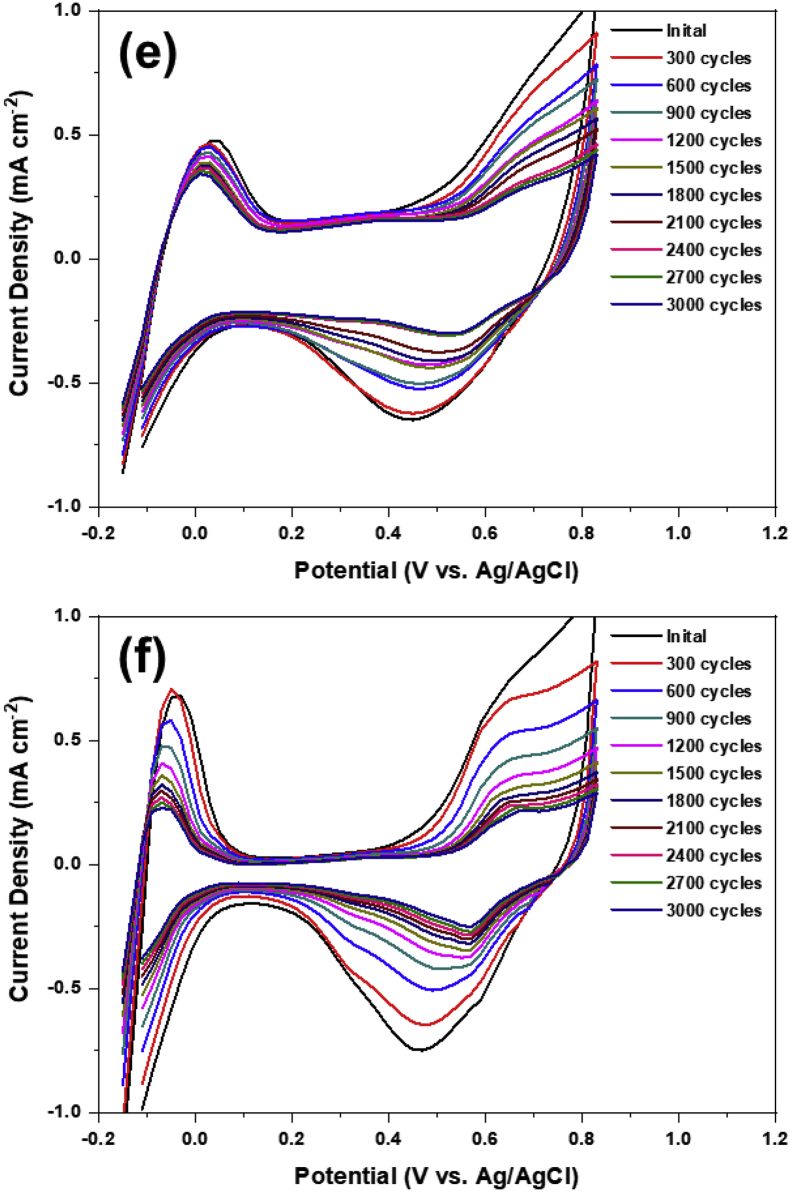
(a) Normalized electrochemical surface area and (b–f) representative CV curves of Au–Pt/C with various molar ratios of Au to Pt; (b) 2:1, (c) 1:1, (d) 0.5:1, (e) 0.33:1 and (f) commercial Pt/C, respectively, during accelerated degradation test up to 3000 cycle.

**Fig. 4:**
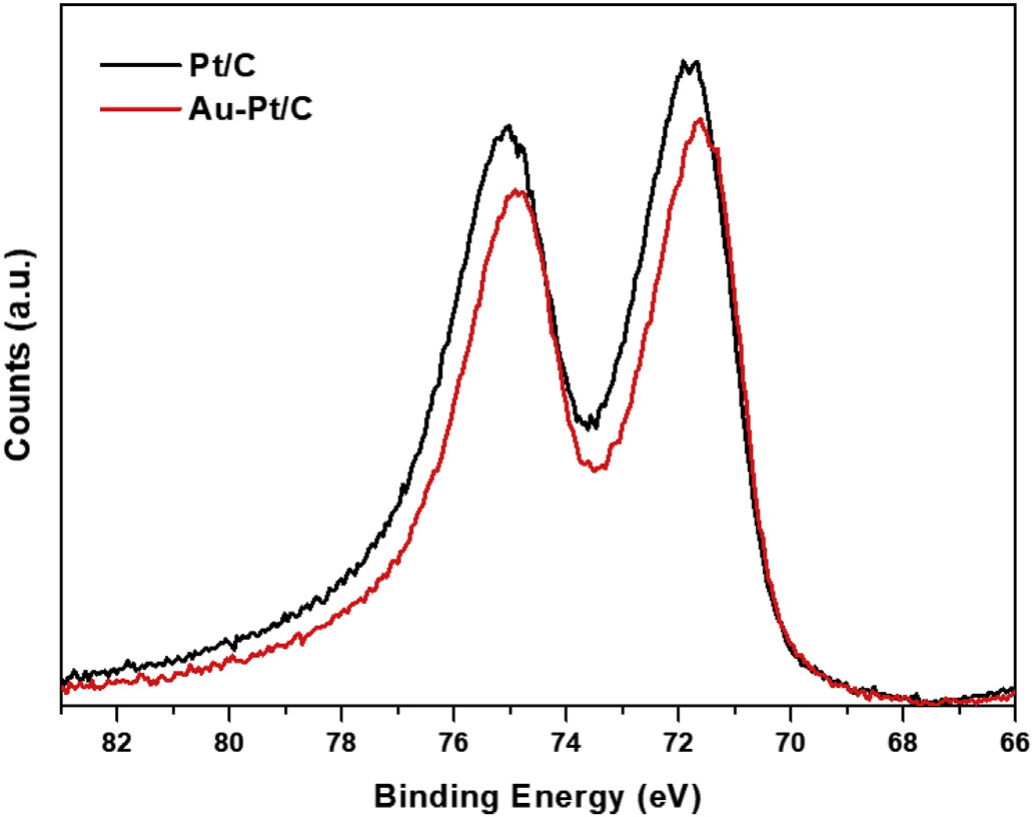
Comparison of X-ray Photoelectron spectra of Pt4f for Au–Pt/C with a molar ratio of 1 to 1 and commercial Pt/C electrode.

The actual ESA are calculated by integrating the hydrogen desorption profile from cyclic voltammogram (CV). The CV curves of Au–Pt/C catalysts with various Au molar ratio and corresponding ESAs are shown in Fig. 2(a) and (b), respectively. The ESAs of Au–Pt/C calculated here are summarized in Table 1. When Au was deposited on Pt/C with Au:Pt = 1:1, the ESA decreased from 62.9 m^2^/g to 50.3 m^2^/g indicating that Au–Pt/C catalyst was successfully synthesized with the form in which Au particles covered the Pt surface. Accelerated degradation tests were carried out for various molar ratio of Au in AuPt/C catalysts as shown in Fig. 3: all catalysts suffered the loss of electrochemical surface area. While commercial Pt/C lost 40% of its original surface area after 3000 potential cycles, Au–Pt/C was more durable to retain 80% of the ESA. The decoration of Pt/C surface with Au could impede the degradation of platinum. In order to further investigate the mechanism of stabilization of Pt incorporated with Au, analysis on chemical state of Pt is carried out with X-ray Photoelectron spectroscopy. (XPS) As shown in Fig. 4, the binding energy of Pt 4f state in Au–Pt/C with a molar ratio of Au to Pt, 1:1 is lower shifted as much as 0.44 eV compared to Pt/C implying that the electron density of Pt is increased by Au atom. In the Au–Pt contact structure, the electron of Au tends to move toward Pt surface due to lower work function of Au than Pt. The abundance of electron of Pt in Au–Pt/C induce the surface of Pt more reductive than that of Pt/C, thus would retard the dissolution of platinum ion from the surface. Therefore, the electronegative characteristics of Pt resulting from incorporation Au might improve the stability of Pt.

**Table 1:** Summary of crystal size, electrochemical surface area, normalized electrochemical surface area on AuPt/C electrodes with molar ratio of Au to Pt, 2:1, 1:1, 0.5:1, 0.33:1 and pristine Pt/C, respectively.

Molar ratio of Au to Pt	Crystal size of Au (nm)	Electrochemical surface Area (m^2^/g)	Normalized ESA (%) after 3000 cycle
Pt/C	–	62.9	59.47
0.33:1 (Au: Pt)	4.9	60.4	72.67
0.5:1	8.5	53.5	70.75
1:1	17.3	50.3	72.57
2:1	23.7	53.9	76.03

**Fig. 5:**
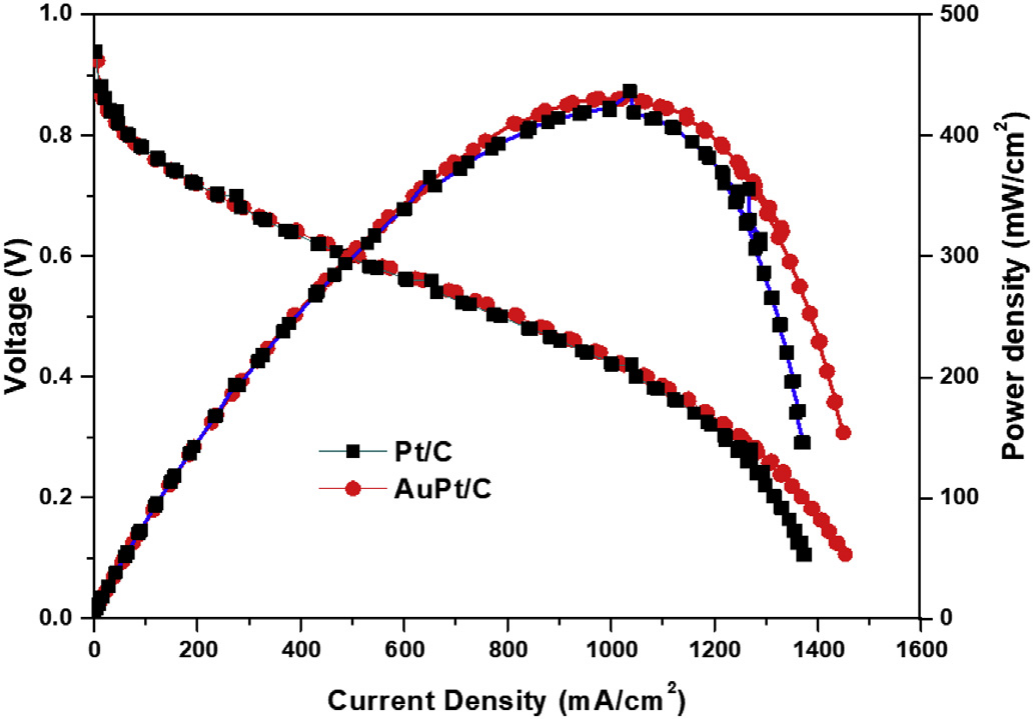
PEMFC performance of Au–Pt/C with a molar ratio of 1 to 1 and commercial Pt/C.

PEMFC performance of Au–Pt/C with a molar ratio of Au to Pt, 1:1 as a ORR (cathode) catalyst was similar to commercial Pt/C (Fig. 5). Considering the electrochemical surface area of Au–Pt/C (50.3m^2^/g) and Pt/C (62.9m^2^/g), Au incorporation on Pt/C surface improved the ORR activity for PEMFC cathode. It is expected that the platinum surface of Au–Pt/C was more reduced than that of commercial Pt/C.

## 2 Experimental design, materials, and methods

### 2.1 Synthesis of Au–Pt/C

Au–Pt/C was synthesized by simple chemical reduction method. In brief, an aqueous solution (100 ml) containing HAuCl4·3H2O (1.0 mM) was added to a given amount of commercial Pt/C (Johnson Matthey 40% Pt) with vigorous stirring at 80 °C. Then, a trisodium citrate solution (10 ml, 38.8 mM) was added to this solution and the resulting mixture was stirred for 15 min. After filtration, the Au–Pt/C was washed three times with water and dried at 70 °C for 24 hours. As increasing the amount of Pt/C catalyst in the synthesis solution, the molar ratio of Au to Pt is controlled from 0.33:1 to 2:1.

### 2.2 Characterization

The crystalline and electronic structures of Au–Pt/C were characterized by powder X-ray diffraction (XRD) and X-ray photoelectron spectroscopy (XPS), respectively. The crystal sizes perpendicular to (220) plane of Pt and Au nanoparticle were calculated from XRD data using the Scherrer equation (Eq. (1)):

(1) $$\text{D}=\frac{K\;\lambda }{\beta \;cos\;\theta }$$

where D is the crystal size, λ is the wavelength of Cu Kα, θ is the diffraction angle of the (220) peak, β is the full width at the half maximum (FWHM), and K is a constant related to crystallite shape, normally taken as 0.9. And, the binding energy of XPS spectrum was corrected by setting the C1s aliphatic signal at 284.8 eV.

### 2.3 Cyclic voltammetry and electrochemical surface area

Cyclic voltammetry were carried out in a typical three-electrode cell with freshly prepared, oxygen free (purged and blanked with Ar gas) 0.5 M H_2_SO_4_ aqueous solution as electrolyte. A platinum wire and an Ag/AgCl (sat. KCl) electrode were used as the counter and reference electrodes, respectively. A Luggin capillary was positioned facing the working electrode at a distance of 5 mm. For the preparation of working electrode, 5 mg of the Au–Pt/C catalyst was wetted with small amount of water, and it was mixed with 1 ml of ethanol and 10 μL of Nafion solution (5 wt%, Sigma Aldrich) in a 10 ml glass bottle. After sufficient stirring and sonication, 30 μL of the catalyst ink was dropped onto a glassy carbon electrode (5 mm dia.) and allowed to dry completely. The cyclic voltammogram (CV) was recorded between −0.2 or −0.15 and 0.8 V (vs. Ag/AgCl) at a scanning rate of 5 mV s^−1^ using a potentiostat/galvanostat. Before the measurement, the potential of the working electrode was cycled for 5 sweeps at 100 mV s^−1^ to electrochemically clean the catalyst surface.

The electrochemical surface area (ESA) was calculated by integrating the final hydrogen desorption profile from the third cycle of three CV scans assuming the value of 210 μC cm^−2^ determined for Pt polycrystalline surface (Eq. (2)).

(2) $$ESA=\frac{Measured\;H\;Charge\;\left(\mu C/cm^{2}\right)}{Pt\;loading\;\left(g/cm^{2}\right)\times 210\mu C/cm^{2}}$$

### 2.4 Accelerated degradation test

The accelerated degradation test of Au–Pt/C catalyst was conducted in the three-electrode cell with the 0.5 M H_2_SO_4_ aqueous electrolyte. The degradation of catalyst was caused by the continuous potential cycling between 0.2 and 0.7 V (vs. Ag/AgCl) at a scan rate of 50 mV s^−1^. During the potential cycling, CV analysis was periodically conducted to monitor the electrochemical surface area of catalyst.

### 2.5 PEMFC performance

The PEMFC performance was measured in a small-scale laboratory cell with an external electrode (2 × 2 cm^2^). The membrane-electrode assembly (MEA) was fabricated by spraying the Au–Pt/C and Pt/C slurries onto cathode and anode sides of Nafion membrane at 50 °C with Pt loading of 0.4 mg cm^−2^. For PEMFC operation, hydrogen and air were introduced into the anode and cathode sides of the PEMFC through a humidifier. The cell performance was tested at 70 °C under 100% relative humidity.
